# Metallothionein immunoexpression in non-syndromic and syndromic keratocystic odontogenic tumour

**DOI:** 10.4317/medoral.20418

**Published:** 2015-04-10

**Authors:** Aline-Cristina-Batista-Rodrigues Johann, Patrícia-Carlos Caldeira, Marcelo-Vidigal Caliari, Ricardo-Santiago Gomez, Maria-Cássia-Ferreira Aguiar, Ricardo-Alves Mesquita

**Affiliations:** 1 DDS, PhD. Pontifícia Universidade Católica do Paraná, School of Health and Biosciences, Curitiba, PR, Brazil; 2 DDS, PhD. DDS, PhD. DDS, PhD. DDS, PhD. Department of Oral Surgery and Oral Pathology, School of Dentistry, Universidade Federal de Minas Gerais; 3 PhD Department of Pathology, Biological Sciences Institute, Universiade Federal de Minas Gerais

## Abstract

**Background:**

To compare the metallothionein (MT) immunoexpression in non-syndromic and syndromic keratocystic odontogenic tumour (KOT), to correlate MT with cellular proliferation, and to evaluate the influence of inflammation in MT.

**Material and Methods:**

Fourteen cases of KOT were submitted to immunohistochemistry for MT and Ki-67 analysis. The lesions were grouped according to their grade of inflammation, and statistical analysis was performed.

**Results:**

MT was higher in non-syndromic KOT than in syndromic KOT (*p*<0.05). No statistical difference in Ki-67 could be identified; however, an inverse correlation was observed between MT and Ki-67 in both lesions. When analysing inflammation, non-syndromic KOT showed no differences in either MT or Ki-67.

**Conclusions:**

The MT immunophenotype of syndromic KOT was different from non-syndromic KOT. MT might not be involved in the proliferation control of both KOT. MT and Ki-67 immunoexpressions proved to be unaffected by inflammation in non-syndromic KOT.

**Key words:** Odontogenic tumours, basal cell nevus syndrome, metallothionein, Ki-67 Antigen, immunohistoche-mistry.

## Introduction

Keratocystic odontogenic tumour (KOT) shows a locally aggressive clinical behaviour associated with a high rate of recurrence. KOT can occur sporadically (non-syndromic KOT), or can be multiple, in association with the Nevoid Basal Cell Carcinoma Syndrome (NBCCS) or Gorlin syndrome (syndromic KOT) (1). The literature describes differences in morphological and immunohistochemical profiles of syndromic and non-syndromic KOT (2-10). These differences have been associated with a higher growth and destructive capacity, as well as with a tendency to develop more recurrences within syndromic KOT (5,8).

Metallothionein (MT) is a low molecular weight protein which major function is related to homeostasis of essential metals. MT is also involved with regulation of cellular differentiation and proliferation - regulating transcriptional factors by donating zinc (11) - and inhibition of cellular apoptosis - by controlling cellular zinc levels through the zinc-dependent antiapoptotic transcription nuclear factor κB, either by inducing antiapoptotic oncogenes or by inhibiting proapoptotic proteins (12-14). This protein can be detected in four isoforms (MT-I to IV). MT-I and MT-II isoforms are similar and can be observed in many tissues, including the oral epithelium (15).

Ki-67 is a nuclear protein correlated with cellular proliferation, present in all active phases of the cell cycle (G1, S, G2, M) but absent in G0. Some studies have shown similar indexes of the immunoexpression of Ki-67 when comparing non-syndromic and syndromic KOTs (16,17), while other studies have reported a higher Ki-67 expression in syndromic KOT (18,19).

In a prior study carried out by the present study’s research team, results showed that the presence of MT in the epithelium of non-syndromic KOT is either less or not related to proliferation (20). On the other hand, it has been hypothesized that differences can be found in MT expression when comparing syndromic and non-syndromic KOT. There is no consensus in the literature whether MT is associated with Ki-67, thus we intended to explore this possibility herein. So, the goals of the present study were to: 1) report and compare the MT immunoexpression in syndromic and non-syndromic KOT, 2) correlate the MT immunoexpression with cellular proliferation in these lesions and 3) evaluate the influence of the inflammation in MT immunoexpression.

## Material and Methods

- Specimens

**Figure 1:**
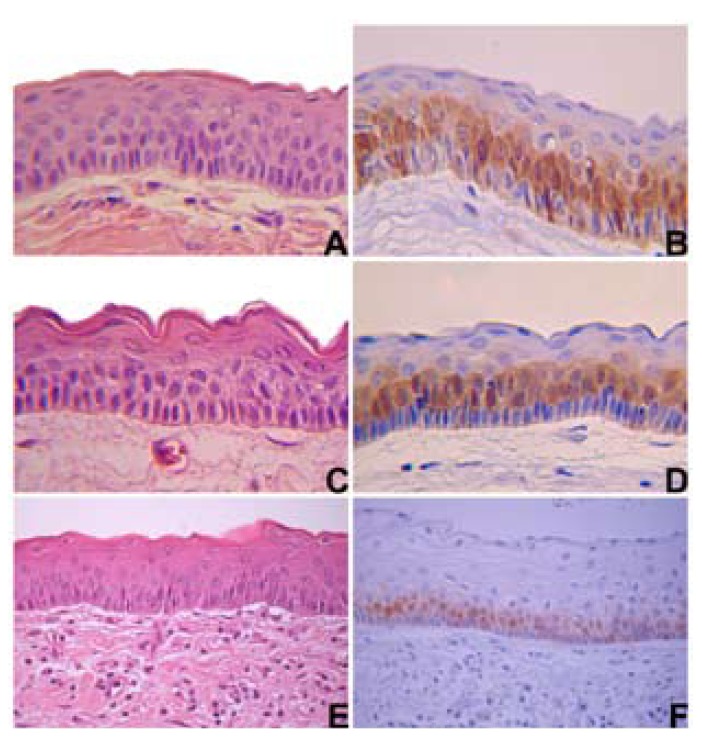
Non-syndromic keratocystic odontogenic tumour: (A) a thin connective tissue lined by stratified squamous epithelium with a well-defined basal layer of palisading columnar or cuboidal cells and with a corrugated surface of parakeratin (Haematoxylin and eosin, X200 original magnification); (B) MT staining was predominantly in nuclei and cytoplasm of the basal and suprabasal keratinocytes (Streptavidin-biotin, X200 original magnification). Syndromic keratocystic odontogenic tumour: (C) similar histological and (D) MT staining than non-syndromic KOT. Inflammed keratocystic odontogenic tumour: (E) histological and (F) MT immunostaining.

Samples diagnosed as KOT: 8 non-syndromic (Fig. 1A) and 6 syndromic (Fig. 1C) cases were retrieved from the files of the Oral Pathology Service of Universidade Federal de Minas Gerais (UFMG). The histological slides stained with haematoxylin and eosin (HE) were reviewed and the diagnoses proved to be in accordance with the 2005 WHO classification (1). Cases of recurrent KOTs and tissue of lesions submitted to decompression were excluded. The study protocol was approved by the UFMG Research Ethics Committee (UFMG/COEP, under protocol number 15/08).

- Inflammatory assessment

Using an optical microscope (Axiolab Zeiss, Oberkochen, Baden-Württemberg, Germany) at 400x magnification, the inflammatory score was determined by counting the inflammatory cells adjacent to the epithelium in 20 consecutive fields, in one high power field-depth from the basement membrane. Inflammation was graded as Grade 0 - no inflammation, Grade 1 - <15 cells/field, Grade 2 - 15-50 cells/field, and Grade 3 - >50 cells/field. The inflammatory score was calculated as the average of all high power fields examined. The KOT samples were divided into two groups according to the inflammatory score: group A - grades 0-2 (mild-to-moderate) and group B - grade 3 (intense) (21).

- Immunohistochemistry

Streptavidin-biotin standard protocol was performed. Sections of 4µm from paraffin-embedded blocks were first deparaffinized and rehydrated, and then submitted to antigen retrieval buffer for 20 minutes at 98ºC. Blockage of endogenous peroxidase activity was performed using 0.3% hydrogen peroxide in all cases. Sections were incubated with primary monoclonal antibodies (MT clone E9; Ki-67 clone MIB-1; Dako Corporation, Carpinteria, CA, USA) and the detection was performed using the LSAB®+system, HRP Peroxidase Kit (Dako Corporation, Carpinteria, CA, USA, K0690) and 3.3’-diaminobenzidine tetra hydrochloride chromogen (DAB, Sigma Chemical, St. Louis, USA, D5637). Mayer’s Haematoxylin was used for counter staining. Squamous cell carcinoma was used as the positive control.

- Immunohistochemical assessment

A digital micro camera (JVC TK-1270/RGB, Tokyo, Japan) was connected to the optical microscope (Carl Zeiss, Axiostar 1122-100, Oberkochen, Baden-Württemberg, Germany). Twenty digital images were taken from each slide using a micro camera at 400x magnification. These images were then analyzed using KS300 software coupled to a Carl Zeiss Image Analyzer (Oberkochen, Baden-Württemberg, Germany).

Firstly, the analysis of MT was performed in basal/parabasal layer and superficial layers separately. However, there was no difference between syndromic and non-syndromic cases considering this stratification. Therefore, further analyses were performed considering MT and Ki-67 imunnoexpression in all layers of the lining epithelium. In addition, the cell compartment with MT staining was categorized as cytoplasmatic and nuclear, cytoplasmatic only or nuclear only, whereas the Ki-67 staining was only nuclear. The indexes of cells labeled for MT and Ki-67 were obtained by dividing the total number of positive cells by the total number of epithelial cells, and multiplying by 100.

- Statistic analysis

BioEstat® 4.0 (BioEstat, Tefé, AM, Brazil) software was used in the statistical analysis. Comparisons between MT and Ki-67 in groups A and B were perfomed only with non-syndromic KOT. The Student t-test was employed in the analysis of total Ki-67, Ki-67 groups A and B, total MT, MT groups A and B, and cytoplasmatic and nuclear MT, as they presented a normal distribution in the Shapiro-Wilk tests. The Mann-Whitney U-test was used to analyze both nuclear and cytoplasmatic MT, as these did not show a normal distribution in the Shapiro-Wilk tests. Statistical significance was accepted at *p*<0.05. Pearson’s correlation was used to evaluate the correlation between MT and Ki-67 in syndromic and non-syndromic KOTs, since they showed a normal distribution. The correlation was graded as: weak – <0.30 moderate – 0.30-0.50; and strong – >0.50.

## Results

MT immunoexpression was identified in epithelial cells in all cases, presenting a mosaic pattern, with cells showing a high staining heterogeneity, from negative to strongly positive. There was no lesion with only nuclear or only cytoplasmic MT expression. All lesions showed MT satining in nucleus and cytoplasm. MT staining was absent or rare in the upper layer and could be easily identified in both the suprabasal and basal layers (Figs. 1B and 1D). In both groups, the staining was predominantly found in nuclei and cytoplasms simultaneously ( Table 1). The mean of MT and Ki-67, and comparisons between the lesions, are displayed in  Table 2.

**Table 1:**
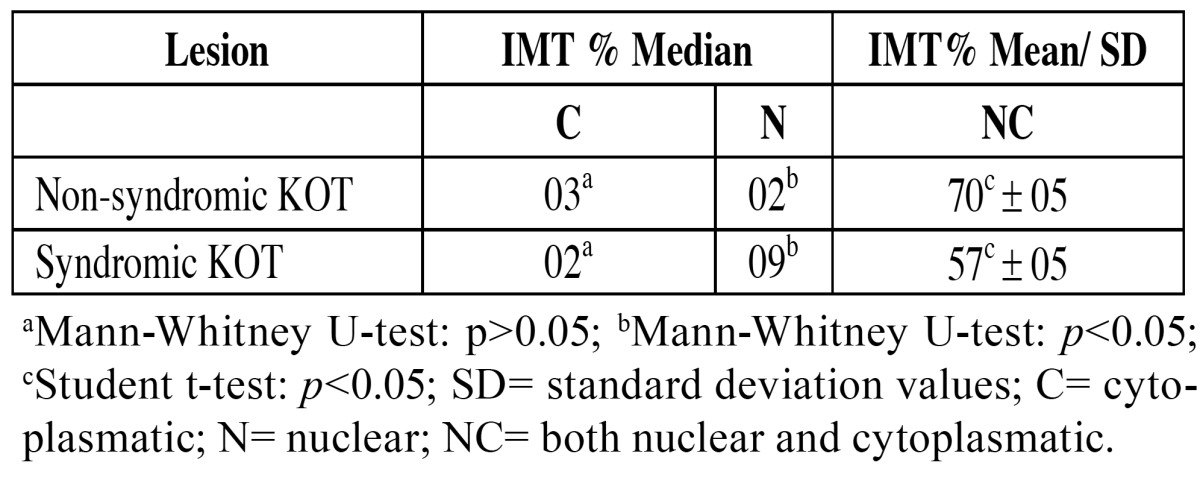
Indexes of cells labeled for metallothionein stratified by cell compartment in non-syndromic KOT and syndromic KOT.

**Table 2:**
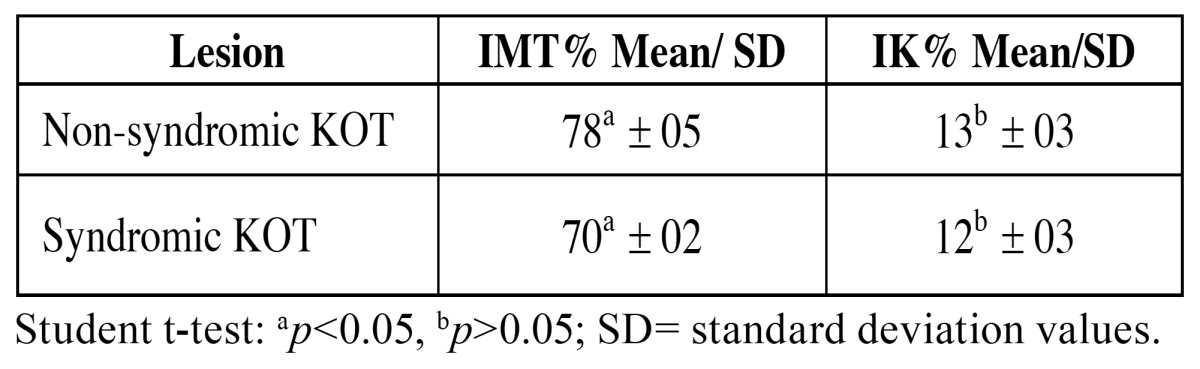
Indexes of cells labeled for metallothionein and Ki-67 in non-syndromic KOT and syndromic KOT.

Statistic tests showed that non-syndromic KOT presented higher MT index than did syndromic KOT (*p*<0.05). This, however, should rather be interpreted as a tendence considering the small sample size, which is a limitation of the present study. Accordingly, further studies should confirm these results. A similar number of Ki-67 positive cells could be observed in non-syndromic and syndromic KOTs. The inverse correlation observed between MT and Ki-67 was strong in non-syndromic KOT (Pearson correlation coefficient [r] = -0.5126) and weak in syndromic KOT (r = -0.1678).

According to the presence and degree of inflammatory infiltrate, in non-syndromic KOT, 4 cases belonged to group A and 4 cases to group B. All cases of syndromic KOT belonged to group A. No statistical difference in MT or in Ki-67 could be observed when comparing group A with group B in non-syndromic KOT.

## Discussion

In mammalian cells, MT is mainly a cytoplasmic protein related to the homeostasis of essential metals, protection against toxicity of heavy metals and free radicals (15), inhibition of cellular apoptosis (13), carcinogenesis (22-24), cellular differentiation, tissue development and cell proliferation (25). Nuclear localization of MT is more closely related to protection against oxidative stress, genomic damage, and genomic regulation of other DNA-related proteins (26). However, in the present study, MT was found mainly in cytoplasmatic and nuclear compartments simultaneously, similar to that reported in previous studies of odontogenic cysts and tumours (20,27). Simultaneous localizalition of MT in nucleus and cytoplasm may well be related to MT functions in various mechanisms of cellular homeostasis.

Apoptosis plays a wide range of roles in tissue development and normal homeostasis, as well as in pathologic conditions (13). MT may inhibit apoptosis by controlling cellular zinc levels through the zinc-dependent antiapoptotic transcription nuclear factor κB, either by inducing antiapoptotic oncogenes or by inhibiting proapoptotic proteins (14). Terminal deoxynucleotidyl transferase-mediated dUTP nick-end labeling (TUNEL) assay and methyl green-pyronin demonstrated that the apoptotic index was slightly higher in syndromic KOT than in non-syndromic KOT, but the difference proved to be insignificant (16). This finding corroborates with results from the present study in which a lower MT index could be observed in syndromic KOT than in non-syndromic KOT (*p*<0.05). The possible participation of MT within apoptotic mechanisms in these lesions should be further elucidated. Moreover, syndromic KOT presents an MT immunophenotype that is different from that found in non-syndromic KOT. It has been suggested that the more aggressive clinical behaviour of syndromic KOT, when compared to non-syndromic KOT, may not be related to differences in cell turnover, proliferation or apoptosis of the epithelial lining, but rather to the multiplicity of lesions and early development of syndromic KOTs (16).

The correlation of the MT and cellular proliferation index varies according to the tissue. In a normal endometrium, MT immunoexpression is inversely correlated with Ki-67 (28), as found in the current study. However, a positive correlation was observed between MT and Ki-67 in malignant lesions (28), whereas no correlation was reported in malignant (22,29), benign and borderline lesions (28). Concerning odontogenic cysts, recent research from the present study’s research group demonstrated diferences in the MT index and cell proliferation in the radicular and dentigerous cysts; however, these findings presented either less or no relation to the KOT or orthokeratinized odontogenic cysts (20). In benign odontogenic tumours, MT may play a role in the stimulation of cell proliferation in solid ameloblastomas and squamous odotogenic tumours (27). By contrast, MT may well inhibit cellular proliferation in the adenomatoid odontogenic tumour (27).

In the present study, the Ki-67 expression proved to be similar in both non-syndromic and syndromic KOTs, which was similar to that reported by other authors (16,17). Other studies have reported a higher Ki-67 expression in syndromic KOT, but these studies used different methods of analysis (18,19). Pan and Li (19) observed that the number of Ki-67+ cells/?m basal membrane in KOTs with PTCH1 mutation was significantly higher than in cases with no PTCH1 mutation. The authors also detected that KOTs harboring PTCH1 truncation-causing mutations showed an even greater Ki-67 immunoexpression than did those with non-truncation-causing mutations, which was also observed when syndromic and non-syndromic KOTs were compared separately (19). These results suggest that PTCH1 mutations, particularly those causing protein truncations, are in fact associated with a subgroup of KOTs, which illustrates an increased cell proliferation index. Further studies correlating PTCH1 mutation and MT immunoexpression in KOTs should be performed to improve scientific knowledge concerning the participation of MT in the behaviour of syndromic or non-syndromic KOT (3).

Metals, hormones, cytokines, a variety of other chemicals, inflammation and stress induce the synthesis of MT (26). Moreover, Kaplan and Hirshberg (30) observed no significant effect of inflammation on the overall Ki-67 expression in KOT. However, in prior studies as well as in the current study, inflammation did not interfere in MT and Ki-67 immunoexpression (20,27).

In conclusion, the MT immunophenotype of syndromic KOT was different from non-syndromic KOT, with lower MT index in syndromic KOT. MT might not be involved in controlling the proliferation of both forms of KOT. MT and Ki-67 immunoexpression proved to be unaffected by inflammation in non-syndromic KOT.

## References

[1] Gorlin RJ (1987). Nevoid basal-cell carcinoma syndrome. Medicine (Baltimore).

[2] Lo Muzio L, Staibano S, Pannone G, Bucci P, Nocini PF, Bucci E (1999). Expression of cell cycle and apoptosis-related proteins in sporadic odontogenic keratocysts and odontogenic keratocysts associated with the nevoid basal cell carcinoma syndrome. J Dent Res.

[3] Kimi K, Kumamoto H, Ooya K, Motegi K (2001). Immunohistochemical analysis of cell-cycle- and apoptosis-related factors in lining epithelium of odontogenic keratocysts. J Oral Pathol Med.

[4] Ohki K, Kumamoto H, Ichinohasama R, Sato T, Takahashi N, Ooya K (2004). PTC gene mutations and expression of SHH, PTC, SMO, and GLI-1 in odontogenic keratocysts. Int J Oral Maxillofac Surg.

[5] Kolár Z, Geierová M, Bouchal J, Pazdera J, Zboril V, Tvrdý P (2006). Immunohistochemical analysis of the biological potential of odontogenic keratocysts. J Oral Pathol Med.

[6] Katase N, Nagatsuka H, Tsujigiwa H, Gunduz M, Tamamura R, Pwint Hp (2007). Analysis of the neoplastic nature and biological potential of sporadic and nevoid basal cell carcinoma syndrome-associated keratocystic odontogenic tumor. J Oral Pathol Med.

[7] González Moles MA, Mosqueda-Taylor A, Esteban F, Gil-Montoya JA, Díaz-Franco MA, Delgado M (2008). Cell proliferation associated with actions of the substance P/NK-1 receptor complex in keratocystic odontogenic tumours. Oral Oncol.

[8] Woolgar JA, Rippin JW, Browne RM (1987). A comparative study of the clinical and histological features of recurrent and non-recurrent odontogenic keratocysts. J Oral Pathol.

[9] Dominguez FV, Keszler A (1988). Comparative study of keratocysts, associated and non-associated with nevoid basal cell carcinoma syndrome. J Oral Pathol.

[10] Brannon RB (1977). The odontogenic keratocyst. A clinicopathologic study of 312 cases. Part II. Histologic features. Oral Surg Oral Med Oral Pathol.

[11] Hecht D, Jung D, Prabhu VV, Munson PJ, Hoffman MP, Kleinman HK (2002). Metallothionein promotes laminin-1-induced acinar differentiation in vitro and reduces tumor growth in vivo. Cancer Res.

[12] Sundelin K, Jadner M, Norberg-Spaak L, Davidsson A, Hellquist HB (1997). Metallothionein and Fas (CD95) are expressed in squamous cell carcinoma of the tongue. Eur J Cancer.

[13] Formigari A, Irato P, Santon A (2007). Zinc, antioxidant systems and metallothionein in metal mediated-apoptosis: biochemical and cytochemical aspects. Comp Biochem Physiol C Toxicol Pharmacol.

[14] Pedersen M, Larsen A, Stoltenberg M, Penkowa M (2009). The role of metallothionein in oncogenesis and cancer prognosis. Prog Histochem Cytochem.

[15] Vasák M (2005). Advances in metallothionein structure and functions. J Trace Elem Med Biol.

[16] Mateus GC, Lanza GH, De Moura PH, Marigo HA, Horta MC (2008). Cell proliferation and apoptosis in keratocystic odontogenic tumors. Med Oral Patol Oral Cir Bucal.

[17] Gurgel CA, Ramos EA, Azevedo RA, Sarmento VA, Silva Carvalho AM, Santos JN (2008). Expression of Ki-67, p53 and p63 proteins in keratocyst odontogenic tumours: an immunohistochemical study. J Mol Histol.

[18] Ba K, LI X, Wang H, Liu Y, Zheng G, Yang Z (2010). Correlation between imaging features and epithelial cell proliferation in keratocystic odontogenic tumour. Dentomaxillofac Radiol.

[19] Pan S, Li TJ (2009). PTCH1 mutations in odontogenic keratocysts: are they related to epithelial cell proliferation?. Oral Oncol.

[20] Johann AC, Caldeira PC, Caliari MV, De Abreu MH, Aguiar MC, Mesquita RA (2011). Metallothionein in the radicular, dentigerous, orthokeratinized odontogenic cysts and in keratocystic odontogenic tumor. Journal of Oral Pathology & Medicine.

[21] Hirshberg A, Lib M, Kozlovsky A, Kaplan I (2007). The influence of inflammation on the polarization colors of collagen fibers in the wall of odontogenic keratocyst. Oral Oncol.

[22] Cardoso SV, Barbosa HM, Candellori IM, Loyola AM, Aguiar MC (2002). Prognostic impact of metallothionein on oral squamous cell carcinoma. Virchows Arch.

[23] Brazão-Silva MT, Cardoso SV, De Faria PR, Dias FL, Lima RA, Eisenberg AL (2013). Adenoid cystic carcinoma of the salivary gland: a clinicopathological study of 49 cases and of metallothionein expression with regard to tumour behaviour. Histopathology.

[24] Gumulec J, Raudenska M, Adam V, Kizek R, Masarik M (2014). Metallothionein - immunohistochemical cancer biomarker: a meta-analysis. PLoS One.

[25] Nishimura H, Nishimura N, Tohyama C (1989). Immunohistochemical localization of metallothionein in developing rat tissues. J Histochem Cytochem.

[26] Cherian MG, Jayasurya A, Bay BH (2003). Metallothioneins in human tumors and potential roles in carcinogenesis. Mutat Res.

[27] Johann AC, Caldeira PC, Souto GR, Abreu MHNG, Aguiar MCF, Mesquita RA (2014). Metallothionein immunoexpression in selected benign epithelial odontogenic tumors. J Oral Pathol Med.

[28] Ioachim EE, Kitsiou E, Carassavoglou C, Stefanaki S, Agnantis NJ (2000). Immunohistochemical localization of metallothionein in endometrial lesions. J Pathol.

[29] Szelachowska J, Dziegiel P, Jelen-Krzeszewska J, Jelen M, Tarkowski R, Wlodarska I (2008). Prognostic significance of nuclear and cytoplasmic expression of metallothioneins as related to proliferative activity in squamous cell carcinomas of oral cavity. Histol Histopathol.

[30] Kaplan I, Hirshberg A (2004). The correlation between epithelial cell proliferation and inflammation in odontogenic keratocyst. Oral Oncol.

